# Drought-Stress Induced Physiological and Molecular Changes in Plants

**DOI:** 10.3390/ijms23094698

**Published:** 2022-04-24

**Authors:** Tomasz Hura, Katarzyna Hura, Agnieszka Ostrowska

**Affiliations:** 1 Polish Academy of Sciences, The Franciszek Górski Institute of Plant Physiology, Niezapominajek 21, 30-239 Kraków, Poland; 2 Department of Plant Breeding, Physiology and Seed Science, Faculty of Agriculture and Economics, Agricultural University, ul. Podłużna 3, 30-239 Kraków, Poland

Soil drought is one of the major abiotic stresses that inhibits the growth, development, and yield of crops all over the world. It is therefore a highly popular research topic that investigated at different levels of plant organization, i.e., molecular, biochemical, physiological, and morphological. This Special Issue, “Drought-Stress Induced Physiological and Molecular Changes in Plants”, presents twelve excellent articles, ten research papers and two reviews, that discuss the latest findings that elucidate the molecular basis of how plants adapt to drought stress. The most interesting results are highlighted, as they may be important for future studies on the recognition of the crucial mechanisms behind drought tolerance. 

The changes in climate that have been observed all over the world indicate a marked trend toward higher temperatures accompanied by reduced annual precipitation [1]. This means that future plants, including crops, will be exposed to increasingly destructive soil droughts. These droughts are exacerbated by the fact that snow cover is often present for only a short period of time and because rain during the vegetation period is rare, and when it does occur, it is of a transient and torrential nature. Despite the development of more accurate weather forecasting models, the unpredictability of the scope, timing, and impact of dry periods on plants still represents a major challenge. Therefore, it is of high importance to recognize the physiological, biochemical, and molecular basis of plant adaptation to extreme soil droughts and to take progressive steppe-formation and the desertification of agricultural, orchard, and horticultural areas into account. 

Plants have developed morphological, physiological, biochemical, and molecular mechanisms to help them acclimatize to dry environments. Although the first three are relatively well recognized, our understanding of the molecular basis of plant drought tolerance, connected with improved water-use efficiency, is still far from complete. The effects of water deficit in plant cells include, e.g., morphological changes, limited growth and development, decreased activity of the photosynthetic apparatus and photosynthetic efficiency, and disturbances in the primary and secondary plant metabolism [2,3,4,5]. 

Plants are capable of avoiding dehydration by means of efficient water uptake (via a well-developed root system), efficient water conduction (increasing the cross-section of the vessels, densely veined leaves, reduced transport distances through the formation of shorter internodes), limiting transpiration (closing stomata with abscisic acid (ABA), increased cuticle thickness, covering the leaves with trichomes, reducing the leaf size or the number of leaves, the reversible folding or curling of the leaf blades, leaf shedding), or water storage (increased succulence). Plants can also tolerate severe dehydration and may go into anabiosis, i.e., a state in which they can cease their metabolic activity almost completely. Plants who are exposed to water stress synthesize and accumulate protective substances, such as dehydrins (proteins with a stable structure under cell dehydration) or carbohydrates that stabilize the phospholipids of the cell membranes. Moreover, they have developed effective repair mechanisms that are initiated when the soil drought subsides [6].

It should be underlined that the above-mentioned adaptation mechanisms that have been developed by plants to combat to soil drought are regulated by multiple genes. It is therefore necessary to identify the key genes that enhance drought tolerance to provide yield stability in global plant production. However, the physiological approach in drought stress experiments still plays an important role in identifying traits and pivotal processes that are under genetic control. Understanding the physiological and molecular basis of plant adaptation to drought will bring about new tools that breeders could use to develop cultivars that are resistant to drought at all of the key stages of growth and, therefore, are better adapted to global climatic changes [7].

In this context, it is our great pleasure to introduce this Special Issue, which focuses on the drought stress that is induced physiological and molecular changes in plants. All twelve papers mainly concentrate on the molecular background of plant acclimatization to drought stress, and each of them represents an interesting approach to this topic. The molecular analyses are supported by biochemical and physiological measurements. It should be pointed out that these studies cover different stages of plant responses to drought stress, from signal perception via target gene expression to functional protein detection. 

Di19 proteins play important roles in abiotic stress responses. An experiment conducted by Wu et al. [8] identified eight Di19 genes in poplar (*Populus* sp.). The plants were treated independently with PEG-6000, NaCl, ABA, and cold. The study provided information on the phylogenetic tree, conserved protein domain, and the structure of Di19 gene members in seven species. The authors demonstrated that *PtDi19-2* and *PtDi19-7* could improve the drought tolerance of transgenic plants through ABA-dependent signaling pathways, which may also be used in poplar breeding practices.

Another published work focuses on the drought-related localization of specific cell wall components, such as glycoproteins, pectins, and hemicelluloses in the root nodules and flower abscission zone in drought-exposed yellow lupine (*Lupinus luteus*) [9]. The immunofluorescence techniques were used to visualize the changes in the cell wall compounds, and the analyses revealed the upregulation of extensins, galactans, arabinans, xylogalacturonan, and xyloglucans. Additionally, the proteins responsible for cell wall remodeling in the root nodules and flower abscission zone in yellow lupine experiencing drought were identified.

Li et al. [10] presented the outcomes of their research concerning the biochemical and cellular mechanisms that are responsible for the regulation of adventitious root (AR) formation under water deficiency in apple trees. The phytohormone content and transcriptomic changes during adventitious root formation were monitored. The study proved that AR formation is a complex biological process that is influenced by multiple hormonal signaling pathways, cell cycle-related genes, and sugar metabolism. The authors indicated the need for further studies on the role of candidate genes in the multiple pathways that are responsible for AR formation.

The study by D’Oria et al. [11] is a report on *Brassica napus* that has been subjected to mild, severe, or severe and extended water deficits. It consists of ionomic, transcriptomic, and metabolic analyses. Surprisingly, the metabolomic and transcriptomic data clearly show that early leaf responses to drought, such as an increase in the abscisic and jasmonic acid content, proline accumulation, and oxidative stress defense, were induced later than the repression of genes related to mineral nutrient transport. The study confirmed the negative impact of drought on the uptake of Mo, Fe, Zn, and Mn by *B. napus* plants.

The article by Baidyussen et al. [12] describes the expression of two specific alleles of zinc-finger transcription factors (ZF-TF), *HvSAP8* and *HvSAP16*, and corresponding SNP markers in barley populations experiencing drought stress. The researchers found that the ZF-TF genes, *HvSAP8* and *HvSAP16*, with different A20/AN1 and AN1/C2H2 domains, exhibited similar trends in their expression profiles in drought-exposed barley. Moreover, single nucleotide polymorphisms (SNPs), which were identified in the upstream regions of the genes, were significantly correlated with changes in their expression profiles. The genotypes with specific alleles at *HvSAP8* and *HvSAP16* indicated a higher 1000 grain weight and more shoots per plant under drought conditions.

Zhang et al. [13] present their results concerning the responses of *Sphagneticola trilobata* (an invasive species), *Sphagneticola calendulacea* (a native related species), and their hybrid to drought stress. They found that *S. trilobata* was the most drought tolerant, and the tolerance of the hybrid did not show heterosis but showed higher tolerance than *S. calendulacea*. *S. trilobata* is capable of rapidly synthesizing large amounts of abscisic acid and reducing stomatal conductance and water loss. The antioxidant capacity of *S. trilobata* was the strongest, while that of *S. calendulacea* was the weakest. The data from the gene expression analysis were in accordance with the physiological data.

The article by Yang et al. [14] focuses on the functional characterization of the *Stipa purpurea P5CS* gene under drought stress. The Δ1-Pyrroline-5-carboxylate synthase (P5CS) is a key enzyme in the proline biosynthetic pathway. The authors reported that the overexpression of *SpP5CS* in *A. thaliana* increased the plant drought resistance and root length and reduced damage to the cell membrane. It is speculated that drought induced *SpP5CS* expression, which increased proline content, improving the drought resistance of the plant.

Jung et al. [15] proved that overexpression of *OsERF83*, a vascular tissue-specific transcription factor gene, confers drought tolerance in rice. Transcriptome analysis revealed that *OsERF83* regulates drought response genes, which are related to the transporter, lignin biosynthesis, terpenoid synthesis, cytochrome P450 family, and abiotic stress-related genes. It was also demonstrated that *OsERF83* up-regulates biotic stress-associated genes. It should be underlined that the findings presented here provide new information on the multiple roles of *OsERF83* in the crosstalk between the abiotic and biotic stress signaling pathways.

The study by Park et al. [16] focused on the *OsERF115*/*AP2EREBP110* transcription factor involved in the tolerance of rice to heat and drought. The authors showed that *OsERF115*/*AP2EREBP110* is a novel positive regulator of combined heat and drought stress and a negative regulator of ABA signaling. They also demonstrated that the enhanced tolerance of *OsERF115*/*AP2EREBP110-OE* plants correlated with better water saving traits under combined heat and drought stress. *OsERF115*/*AP2EREBP110* is suggested to be a candidate gene for genetic engineering solutions to develop heat- and drought-tolerant crops.

Another research paper focused on a genome-wide approach to identify quantitative trait loci (QTL) for drought tolerance in tetraploid potato (*Solanum tuberosum* L.) [17]. QTLs analyses were performed in cultivated potatoes to determine the drought tolerance index, tuber starch content, tuber starch yield, tuber fresh weight, and selected transcripts and metabolites under control and drought treatments. The authors underline that this is the first report using the new SSR-primer combinations derived from candidate genes for drought tolerance to construct genetic maps in tetraploid potatoes.

In their review, Hasan et al. [18] discussed γ-aminobutyric acid (GABA) as a key player in plant drought stress resistance. They highlighted that under drought stress, GABA increases the water-use efficiency or photosynthesis performance and activity of antioxidant enzymes and reduces ROS production, MDA, and H_2_O_2_ content while also protecting the cell membrane. The authors suggest that GABA may play a significant role in the closing and opening of plant stomata. They also list further research directions that may help to elucidate the role of γ-aminobutyric acid in plant adaptation mechanisms to soil drought.

The review by Hrmova et al. [19] discusses the plant transcription factors (TFs) involved in drought and associated stresses. TFs play a significant role in signal transduction that spans the perception of a stress signal and the expression of stress responsive genes. Seven interrelated sections of this review organize and systematize our current and broad knowledge on the role of TFs in drought stress. The authors underline the importance of TFs and suggest the areas that future studies should focus on to provide accurate molecular descriptions of TF functions to understand their specific biological roles.

In summary, global climate change brings about extreme droughts and enhances steppe-formation and the desertification of agricultural, orchard, and horticultural areas. Hence, it is critical to understand the molecular basis of plant responses to soil drought. This Special Issue presents the results of the most recent studies on the physiological and molecular changes in plants experiencing drought stress. The published papers cover a wide range of research topics. Readers will find examples using modern research methods, interesting experimental setups, and open-ended questions that may inspire them to take up innovative research directions. The manuscripts gathered in this issue shed new light on the molecular mechanisms that allow plants to adapt to drought stress and significantly contribute to improving our knowledge in this field.

## Data Availability

No data were generated for the present article.
